# Application of novel non-invasive ophthalmic imaging to visualize peripapillary wrinkles, retinal folds and peripapillary hyperreflective ovoid mass-like structures associated with elevated intracranial pressure

**DOI:** 10.3389/fneur.2024.1383210

**Published:** 2024-06-18

**Authors:** Michaela Graven-Nielsen, Alfredo Dubra, Robert L. Dodd, Steffen Hamann, Heather E. Moss

**Affiliations:** ^1^Department of Ophthalmology, Stanford University, Palo Alto, CA, United States; ^2^Department of Ophthalmology, Rigshospitalet, Glostrup, Denmark; ^3^Department of Neurosurgery, Stanford University, Palo Alto, CA, United States; ^4^Department of Clinical Medicine, University of Copenhagen, Copenhagen, Denmark; ^5^Department of Neurology and Neurological Sciences, Stanford University, Palo Alto, CA, United States

**Keywords:** idiopathic intracranial hypertension (IIH), pseudotumor cerebri syndrome, ophthalmic imaging, optical coherence tomography (OCT), adaptive optics scanning light ophthalmoscopy (AOSLO), retinal folds, peripapillary wrinkles, peripapillary hyperreflective ovoid mass-like structure (PHOMS)

## Abstract

**Background:**

Elevated intracranial pressure (ICP) is a serious and potentially life-threatening condition, for which clinically useful non-invasive measures have been elusive, in some cases due to their inadequate sensitivity and specificity. Our aim was to evaluate novel non-invasive ophthalmic imaging of selected pathological features seen in elevated ICP, namely peripapillary hyperreflective ovoid mass-like structures (PHOMS), peripapillary wrinkles (PPW) and retinal folds (RF) as potential biomarkers of elevated ICP.

**Methods:**

This single-center pilot study included subjects with untreated or incompletely treated high ICP. The retinas of these subjects were evaluated with averaged en-face optical coherence tomography (OCT), OCT retinal cross-sections (OCT B-scans), adaptive optics scanning light ophthalmoscopy (AOSLO), and fundus photos.

**Results:**

Seven subjects were included in the study. 6 subjects with high ICP (5 idiopathic intracranial hypertension, 1 medication induced, 30.8 ± 8.6 years, 75% female, 5 with papilledema) and 1 control (20–25 years) were included. PHOMS, PPW and RF were present in all subjects with papilledema, but neither in the high ICP subject without papilledema nor in the control subject. Averaged en-face OCT scans and AOSLO were more sensitive for PPW and RF than OCT B-scans and commercial fundus photos.

**Conclusion:**

PPW, RF and PHOMS volume have potential as non-invasive biomarkers of ICP. Novel imaging modalities may improve sensitivity. However, lack of automated image acquisition and processing limits current widespread adoption in clinical settings. Further research is needed to validate these structures as biomarkers for elevated ICP and improve clinical utility.

## 1 Introduction

Pseudotumor cerebri (PTC) syndrome is a condition of elevated intracranial pressure (ICP). This umbrella term can be divided into primary PTC, with idiopathic intracranial hypertension (IIH) as a subset within this group, and secondary PTC (SPTC), when the elevated intracranial pressure (ICP) is precipitated by a secondary cause, such as, venous sinus thrombosis, medications, or non-oncologic medical conditions (1). PTC is diagnosed using the Modified Friedman Criteria (1) or the Modified Dandy Criteria (2, 3). These criteria include the evaluation of ICP, cerebrospinal fluid composition, and symptoms of elevated ICP—often including papilledema—and a normal neurologic examination except for cranial nerve abnormalities. Elevated ICP is most frequently established using lumbar puncture opening pressure (LOP)—an invasive procedure with the most common complication being post-dural puncture headache (4, 5). LOP is often performed only at the time of diagnosis, and treatment decisions are based on indirect evidence including patient symptoms. The result is highly dependent on the positioning of the patient, the patient’s ability to relax, and the expertise of the physician performing the procedure (1, 4, 6). Thus, there is a significant unmet need for non-invasive and precise surrogate measures of ICP, for both early diagnosis and monitoring of elevated ICP. Previous studies have investigated potential non-invasive methods, for example transorbital ultrasound measuring the optic nerve sheath diameter, otic methods using inner- and outer-ear responses to ICP changes, the use of Doppler technology for measuring cerebral- and ophthalmic artery blood flow velocity, and radiological approaches. The limitation of these methods, however, means that they have not replaced or been able to reliably supplement LOP (6).

A possible consequence of elevated ICP is vision loss (7, 8) due to damage to the axons of the retinal ganglion cells (RGC) which form the optic nerve and the retinal nerve fiber layer (RNFL). This impact can be visualized by examination of the optic nerve head (ONH), which can be swollen (papilledema) and/or atrophic. The damage can be quantified through several techniques, commonly using optical coherence tomography (OCT), specifically through the measurement of peripapillary RNFL thickness and macular ganglion cell complex (GCC) thickness (9, 10). Although fundus photos and OCT are helpful in the management of PTC, both pose challenges when assessing the severity of optic disc edema and relating this to ICP. On fundus photography, optic disc edema is most often evaluated based on Frisén grade (11), which is known to suffer from poor interrater reliability (12). On OCT, optic disc edema is easier to visualize and quantify, but severe optic disc edema can affect metrics due to poor image quality (e.g., blurry images, or difficulty capturing the entire ONH) and inaccurate image segmentation (13, 14).

Ophthalmic imaging can also be used to visualize other consequences of elevated ICP on ocular anatomy. Peripapillary wrinkles (PPW; also known as “Paton’s folds”) and retinal folds (RF) are findings associated with papilledema, thought to be caused by biomechanical stress on the retina, and can be visualized with OCT as well as fundus photography (15, 16). Peripapillary hyperreflective ovoid mass-like structures (PHOMS) have been established as a common but nonspecific marker of axoplasmic stasis causing peripapillary axonal distension and are seen accompanying several conditions characterized by axoplasmic stasis, including papilledema (17, 18).

Some newer OCT approaches have not yet been studied in papilledema. For example, averaging en-face OCT scans facilitates visualization of cellular level features such as hyalocytes by improving signal-to-noise ratio (19). Hyalocytes are mononuclear macrophages of the vitreous body that can be found close to the inner limiting membrane (ILM) [an average of 50 μm from the inner surface of the retina (20)] as hyperreflective white stellate structures (19). These cells have a dynamic nature and have been shown to change in terms of cell morphology and motility in response to injury and, thus, may be relevant to papilledema (21–23).

A state-of-the-art technique for measurement of structural retinal changes and afferent visual pathway damage that has not been studied in papilledema is adaptive optics (AO) coupled with OCT or scanning laser ophthalmoscopy (SLO). AO ophthalmoscopy can achieve cellular level resolution of the retina (24), and detect pathological alterations before clinical symptoms appear (25), achieving this by correcting for aberrations in ocular optics during image acquisition (26, 27). AOSLO and AO-OCT have been applied to visualize microstructural changes of photoreceptors (24, 25, 28), retinal cellular behavior (e.g., macrophages and microglia) (24, 29), blood flow (24, 25) and individual nerve fiber bundle structure and function, but have not previously been applied in papilledema (24–27).

The purpose of this pilot study was to apply conventional and averaged OCT imaging as well as AOSLO imaging to advance the characterization of ocular changes in subjects with IIH/SPTC, focusing on PPW, RF and PHOMS, and to assess their candidacy as potential biomarkers for elevated ICP.

## 2 Methods

This was a prospective observational pilot study approved by the Institutional Review Board at Stanford University. The study adhered to the tenets of the Declaration of Helsinki. Written informed consent was obtained from all subjects prior to participation.

### 2.1 Subjects

Seven subjects were included in the study. Six subjects were recruited from the Byers Eye Institute, Department of Ophthalmology at Stanford University from November 2022 to June 2023. Inclusion criteria were: Age ≥ 18 years with untreated or incompletely treated IIH or SPTC. Screening was performed by the attending neuro-ophthalmologist. Subjects were defined as incompletely treated if they had begun treatment at time of enrollment, but still had symptoms and/or signs of elevated ICP. Subjects were defined as untreated if they had not begun medical or surgical treatment at the time of enrollment. A control subject was recruited from Spencer Center for Vision Research at Stanford University. Patients and controls were excluded if they had neurological or ophthalmic conditions apart from refractive errors and non-visually significant cataracts. All subjects underwent baseline imaging. Follow-up imaging was conducted on one subject 2 ½ months after the baseline visit.

### 2.2 Medical history, visual field, visual acuity, intraocular pressure and refraction

Blood pressure and pulse were measured with a standard blood pressure cuff at the study visit. Intraocular pressure (IOP) was measured at the study visit using a Tono-Pen (Reichert Technologies, Depew, NY, United States). Data on refraction errors, perimetric mean deviation (MD; Automated perimetry, Humphrey Visual Field (HVF) Analyzer. Either 30-2 or 24-2) and best corrected visual acuity (VA) using a Snellen chart was collected. The values were obtained from the patient’s most recent appointment with their attending physician as documented in the electronic health record or were measured at the study visit. Vision loss was categorized based on HVF MD (MD >−2 dB = normal vision, MD −2 to −6 dB = mild vision loss and MD < −6 dB = moderate/severe vision loss). For VA the best value for each eye was converted to LogMAR values (logarithm of minimum angle of resolution). Medical history was collected from the patient charts and supplemented by interview during the study visit.

### 2.3 Image acquisition

Pupil dilation was performed prior to image acquisition in 6 of 7 subjects.

OCT imaging was performed on all subjects, according to a scanning protocol developed by the Optic Disc Drusen Studies Consortium, using the Spectralis OCT (Heidelberg Engineering, Heidelberg, Germany). This protocol includes (1) a dense optic nerve head volume (ONH) scan in enhanced depth imaging mode (EDI), averaging 30 B-scans, (2) a 24-line radial ONH scan in EDI mode, (3) a peripapillary scan for evaluation of RNFL thickness, and (4) a macular volume scan for evaluation of the macular layers (30).

En-face OCT images of the PPW/RF and hyalocytes anterior to the ILM were generated by capturing volumetric images using the RTVue-XR Avanti (OptoVue/Visionix US, Lombard, Illinois, USA). The protocol consists of 10 volume scans of a 3.0 mm^2^ retinal area over the macula and a 4.5 mm^2^ centered over the ONH (22). Fewer scans (3 or more) were captured in two of the subjects.

AOSLO images of the nerve fiber layer were captured using a custom instrument (31) in 3 subjects—2 with elevated ICP and the control subject. During imaging, the subject was in a seated position and using a bite bar for stability, while fixating their gaze on a target. Short videos with a 1–2-degree field of view were captured. Axial length of the eye was obtained using the ocular biometry HP-OCT (Cylite, Mulgrave, Victoria, Australia) in order to calculate a retinal magnification factor in AOSLO images.

The most recent widefield fundus photos (Optos) taken at the outpatient clinic were collected from the patient charts.

### 2.4 Image analysis

#### 2.4.1 Conventional measurements

Structural OCT segmentation (Spectralis) was performed automatically using commercial software included with the device (HEYEX 2, Heidelberg Eye Explorer, Heidelberg Engineering) and manually corrected. Images were excluded if the quality of the image was <24 or if substantial artifacts were noted. For subjects with multiple images of one location, the image with the highest quality was chosen. Global peripapillary RNFL thickness was extracted from the peripapillary ring scan. Central ONH thickness, maximum height centrally in the ONH and central ONH volume were extracted within a 1 mm diameter circle centered on the optic nerve head (Supplementary Figure 1).

Ganglion cell atrophy was assessed using average GCC thickness, which is the sum of RNFL thickness, ganglion cell layer (GCL) thickness, and inner plexiform layer (IPL) thickness, in the foveal and parafoveal regions on the macular OCT volume scan.

The Frisén grade on the fundus photos was evaluated by two investigators (H.E.M and M.G.N).

#### 2.4.2 Peripapillary wrinkles, retinal folds, and hyalocytes

The presence and morphology of PPW&RF in the retina was explored using a published image analysis protocol previously applied to hyalocyte analysis (21, 32, 33). Full superficial vascular OCT-angiography (OCT-A, Optovue) slabs between the ILM and 9 microns below the IPL at the macula region, and en-face OCT-reflectance (OCT-R) slabs extending from the ILM to 3 or 6 microns above at the ONH and macula region were extracted using commercial device software (RTVue, Optovue). The distance above ILM on the OCT-R slabs was determined based on image contrast and clarity. Only images with quality ≥6 were included. The image with the highest contrast and the least motion artifact was selected as the reference image. The en-face OCT-R slabs for both regions were registered to this reference image using the Register Virtual Stack Slices plug-in on Fiji (ImageJ, National Institute of Health, Bethesda, Maryland, United States). Further processing of the macula required applying the set of vascular OCT-A images to the corresponding OCT-R slabs using the Transform Virtual Stack Slices plug-in in Fiji. Using this processing protocol, the images were aligned and averaged to reduce motion artifacts and improve contrast. At least 3 images were averaged to generate the final averaged en-face OCT image.

Presence of PPW and RF was assessed by review of the averaged en-face OCT scans following the nomenclature developed by Sibony et al. (15). PPW are described as “fine, closely spaced folds” on the disc surface or within half a disc diameter onto the peripapillary retina measured from the edge of the edema/disc. The PPW could be concentric (follow the curvature of the ONH) or spiral (radiate obliquely out from the ONH). RF were defined as “periodic surface or intraretinal undulations,” with a larger peak-to-peak distance than PPW, greater than half a disc diameter from the disc/edge of the edema. RF were also categorized according to their orientation toward the ONH or macula: Radial, oblique or horizontal. In cases where the categorization of the PPW/RF on the averaged en-face OCT scans was not clear, we compared the scans to the OCT B-scans. When PPW/RF visualized on the averaged scans were not visible on the B-scans, they were classified as B-scan negative PPW or RF. Depending on the location and orientation of the PPW/RF, the same averaged en-face OCT image could have both B-scan positive and B-scan negative PPW/RF. The averaged en-face OCT scans were also qualitatively evaluated for the distribution and location of hyalocytes.

#### 2.4.3 Peripapillary hyperreflective ovoid mass-like structures

A PHOMS extends as a full or partial torus (“donut” shape) circumferentially around the Bruch’s membrane opening (BMO). PHOMS volumes were calculated based on measurements performed on radial EDI-OCT B-scans as previously described in detail (18). Briefly, in 6 of 24 radial EDI-OCT B-scans (each B-scan being 30 degrees apart) we evaluated the presence of PHOMS fragments. If present, the fragments were identified on either one or both sides of the Bruch’s membrane opening (BMO). Horizontal and vertical radii of the individual fragments, the diameter of the BMO (Table 1) and the distance from the outermost edge of the BMO to the point of intersection between horizontal and vertical radii were manually measured using tools in the commercial device software. BMO was measured as the distance between the two opposing margins of Bruch’s membrane on the B-scans (Supplementary Figure 2). Utilizing these values, PHOMS volume was calculated using the geometric formula for the volume of a torus (18, 34). If the presence of PHOMS could not be evaluated on a B-scan image due to artifact or motion the next clockwise adjacent scan with no artifact was used. In cases with severe edema the PHOMS were approximated as an ellipse with a peripapillary location above Bruch’s membrane. In some cases with high artifact or shadowing, PHOMS measurements were not conducted. If there was uncertainty regarding the measurement of PHOMS in a given scan an independent investigator reviewed these scans.

**Table 1:** PHOMS visualization and measurements.

Subject		Volume [mm^3^]	Location	Bruch’s membrane opening^1^ [μm]	Challenges	Frisén grade
1	OD	2.78	Full torus	1648.83	–	2
	OS	2.15	Partial torus	1691.33	Could not be measured in 1 B-scan	2
2	OD	0.00	Absent	1343.33	Drusen	0
	OS	0.00	Absent	1285.83	–	0
3 (Baseline)	OD	–	Full torus	1608.67	Could not be measured due to the severity of the papilledema	3
	OS	–	Full torus	1668.33	Could not be measured due to the severity of the papilledema	4
3 (Follow-up)	OD	0.25	Partial torus	1605.50	–	1
	OS	0.53	Partial torus	1614.33	Vessel obstructing 1 B-scan	1
4	OD	2.08	Partial torus	1679.17	Could not be measured in 2 B-scans	4
	OS	1.35	Partial torus	1617.83	Could not be measured in 1 B-scan	2
5	OD	1.27	Partial torus	1418.00	Could not be measured in 2 B-scans	1
	OS	0.91	Partial torus	1479.67	Could not be measured in 2 B-scans	1–2
6	OD	1.11	Partial torus	1596.33	–	2
	OS	1.39	Partial torus	1516.33	–	2
Control	OD	0.00	Absent	1483.00	–	0
	OS	0.00	Absent	1357.00	–	0

OD, Right eye; OS, Left eye. ^1^The average of the diameter of the Bruch’s membrane opening measured on the 6 transverse B-scans on the radial scan.

#### 2.4.4 Adaptive optics scanning light ophthalmoscopy

The AOSLO videos were registered using custom software (35) to create high signal-to-noise ratio confocal and non-confocal split detection retinal images (36). The registered images were manually tiled using Adobe Photoshop (Adobe, San Jose, California, United States) and Fiji (22). The retinal areas were then qualitatively evaluated for the presence of structural changes of the NFL, signs of axoplasmic stasis, and other potentially pathologic findings attributable to high ICP.

## 3 Results

### 3.1 Subject demographics

Six subjects with elevated ICP (5 IIH, 1 SPTC due to minocycline) were enrolled in the study (4 female, 1 male and 1 transgender male, age range 18 to 41 years, mean age of 30.83 ± 8.59; Table 2). All high ICP subjects had LOP ≥ 25 cm H_2_O. 2 subjects were untreated at the time of imaging and 4 were incompletely treated with acetazolamide (ACZ). 5 subjects had papilledema at the time of imaging, while one did not. One subject returned for follow-up imaging 2 ½ months after baseline imaging. This subject was incompletely treated at baseline imaging and continued treatment with ACZ in the period between the study visits. 1 control subject was enrolled (20–25 years old; Table 2). The control had no known ophthalmological conditions apart from a refractive error and no neurological conditions.

**Table 2:** Subject baseline characteristics and demographics.

Subject	1	2	3	4	5	6	Control
Age range (enrollment)	36–40	40–45	36–40	30–35	18–22	20–25	20–25
Ethnicity	Non-Hispanic/-Latino	Hispanic/-Latino	Non-Hispanic/-Latino	Non-Hispanic/-Latino	Hispanic/-Latino	Non-Hispanic/-Latino	Non-Hispanic/-Latino
Race	Unknown	White	Asian	White, Black	Black	White	White
Diagnosis	IIH	IIH	IIH	IIH	SPTC (minocycline)	IIH	Control
LOP [*cm H_2_O*] (at diagnosis)	32	30	39	45	39	52	N/A
Symptoms within 3 months of study visit^1^	1	1, 2, 4, 6	1, 3, 4, 5 + fatigued	1, 2, 3	1, 2, 5, 6	1, 2, 4	No symptoms
Treatment status at study visit	Untreated	Incompletely treated with ACZ for 11 weeks 4 days.	Baseline: Incompletely treated with ACZ for 5 weeks 5 days (40 days)	Incompletely treated with ACZ for 21 days	Incompletely treated with ACZ for 20 days	Untreated	N/A
		Off ACZ for 6 weeks 6 days before visit.	Follow-up: Incompletely treated with ACZ for 11 weeks 4 days (since commencement of treatment)				
Time between diagnosis and baseline study visit	8 days	4 months 17 days	40 days (baseline)	22 days	20 days	20 days	N/A
Diagnosis confirmation tests	LP, MRI	LP, MRI	LP, MRI	LP, MRI	LP, MRI	LP, MRI	N/A

^1^Symptoms: 1, Headache; 2, Tinnitus; 3, Transient vision loss; 4, Blurred vision; 5, Double vision; 6, Nausea and vomiting. IIH, Idiopathic intracranial hypertension; SPTC, Secondary pseudotumor cerebri syndrome; LOP, Lumbar puncture opening pressure; MRI, Magnetic resonance imaging; LP, Lumbar puncture; ACZ, Acetazolamide.

### 3.2 Optic nerve structure and visual function

Clinical ophthalmic assessment measurements are summarized in Table 3. All subjects had excellent central vision and normal to mildly impaired peripheral vision. None of the subjects had a refractive error exceeding spherical equivalent of −2.88 for myopic and 1.25 for hyperopic subjects. The intraocular pressure of all subjects was within normal range (12–21 mmHg).

**Table 3:** Visual function and conventional OCT measurement.

Subject		VA (LogMAR)	HVF	Peripapillary RNFL thickness, *(global)* [μm]	Central ONH thickness, *maximum* [μm]	Central ONH thickness, *average* [μm]	Central ONH volume [mm^3^]	Frisén grade	GCC thickness [μm]
1	OD	0	Normal	196	1,194	888	0.7	2	111.78
	OS	0	Normal	163	922	547	0.43	2	111.89
2	OD	0.1	Normal	110^*^	666	456	0.36	0	118.23
	OS	0.1	LR	103	749	453	0.36	0	121.67
3 (Baseline)	OD	0	Normal	174	823	517	0.41	3	96.67
	OS	0	Normal	198	875	631	0.5	4	97.00
3 (Follow-up)	OD	−0.1	NP	109	611	355	0.28	1	97.78
	OS	0	NP	115	598	425	0.33	1	96.56
4	OD	0.1	NP	147	1,075	754	0.59	4	PS
	OS	0	NP	117	793	367	0.29	2	PS
5	OD	0	Mild vision loss	217	809	545	0.43	1	101.23
	OS	0	Mild vision loss	209	940	590	0.46	1–2	102.23
6	OD	0	Mild vision loss	149	674	393	0.31	2	107.67
	OS	0	Mild vision loss	184	809	471	0.37	2	104.67
Control	OD	−0.1	Normal	105	438	234	0.18	0	103.00
	OS	−0.1	Normal	103	446	245	0.19	0	102.00

OD, Right eye; OS, Left eye; RNFL, Retinal nerve fiber layer; HVF, Humphrey visual field; LR, Low test reliability; VA, Visual acuity; ONH, Optic nerve head; GCC, Ganglion cell complex; PS, Poor segmentation; NP, Not performed. ^*^Optic disc drusen was present.

Subject 2 was found to have an optic disc drusen in the right eye, which could affect the RNFL thickness on this eye (Supplementary Figure 3).

### 3.3 Peripapillary wrinkles and retinal folds

**Figure 1:**
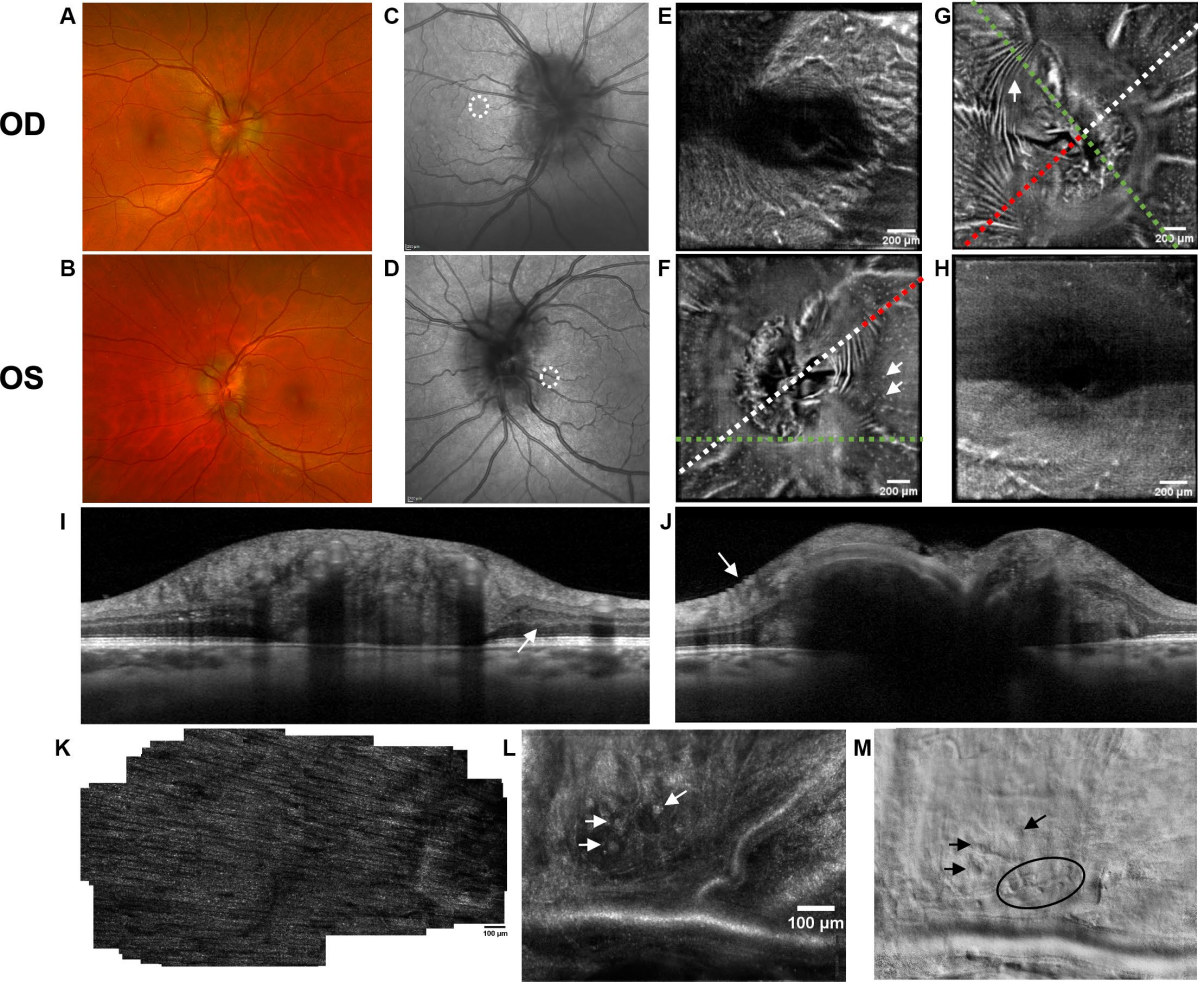
Retinal imaging of subject 1 with IIH. **(A)** Color fundus photo OD. **(B)** Color fundus photo OS. **(C)** SLO image of the OD ONH. The white circle indicates the region where image **(K)** was captured with the AOSLO. **(D)** SLO image of the OS ONH. The white circle indicates the region where images **(L,M)** were captured with the AOSLO. **(E)** Averaged en-face OCT scan of the OD macula. **(F)** Averaged en-face OCT scan of the OS ONH with hyalocytes (white arrows). B-scan negative RF were identified in the transverse section indicated by the white dashed line, specifically at the location of the red dashed line. The green dashed line indicates the transverse position of the B-scan in image **(I)**. **(G)** Averaged en-face OCT scan of the OD ONH with PPW (white arrow). The green dashed line indicates the transverse position of the B-scan in image **(J)**, where the same PPW can be visualized (white arrow). The red dashed line indicates the specific location of the B-scan negative PPW inferotemporal to the ONH on the transverse B-scan indicated by the white dashed line. **(H)** Averaged en-face OCT scan of the OS macula. **(I)** EDI-OCT B-scan of the OS ONH with peripapillary intraretinal folds (white arrow). The green dashed line in image **(F)** indicates the transverse position on the en-face image. The intraretinal folds cannot be visualized on image **(F)**. **(J)** EDI-OCT B-scan of the OD ONH with PPW (white arrow). The green dashed line in image **(G)** indicates the transverse position on the en-face image. Due to papilledema PHOMS could not be measured precisely in this scan. **(K)** Confocal AOSLO image of the retinal nerve fiber layer on OD [region is shown in image **(C)**]. **(L)** Confocal AOSLO image of the retinal nerve fiber layer close to the optic disc on OS [region is shown in image **(D)**]. The white arrows indicate hyperreflective dots with a cellular appearance. **(M)** Non-confocal split detection AOSLO image of the retinal nerve fiber layer on OS [region is shown in image **(D)**]. The black arrows indicate the same hyperreflective dots seen on image **(L)**. The black circle outlines other potential cellular features.

**Figure 2:**
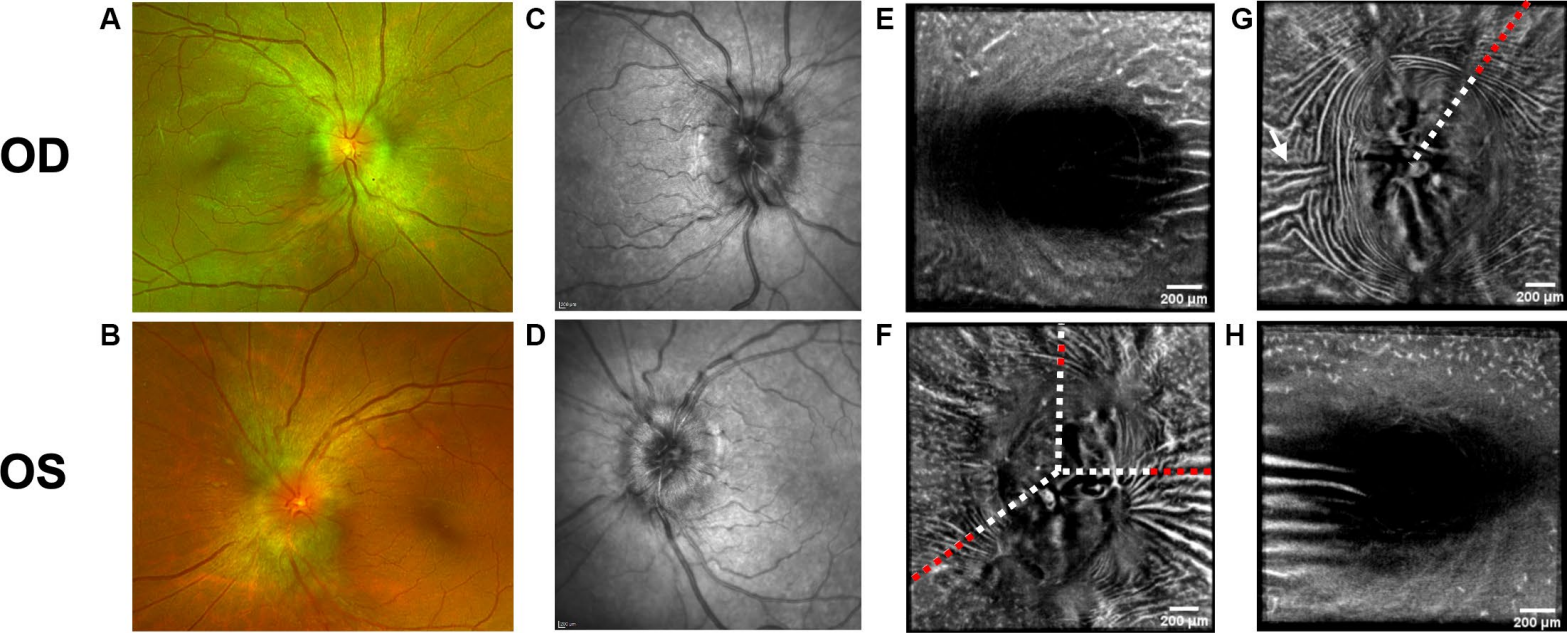
Baseline retinal imaging of subject 3 with IIH. **(A)** Color fundus photo OD. **(B)** Color fundus photo OS. **(C)** SLO image of the OD ONH. **(D)** SLO image of the OS ONH. **(E)** Averaged en-face OCT scan of the OD macula. **(F)** Averaged en-face OCT scan of the OS ONH. The white dashed lines indicate the transverse sections, where B-scans have been captured. The red dashed lines indicate the locations of B-scan negative RF and PPW in these transverse sections. **(G)** Averaged en-face OCT scan of the OD ONH with horizontal retinal folds in the papillomacular bundle (white arrow). The red dashed line indicates the location of B-scan negative PPW and RF on the transverse B-scans, whose location is indicated by the white dashed line. **(H)** Averaged en-face OCT scan of the OS macula.

**Figure 3:**
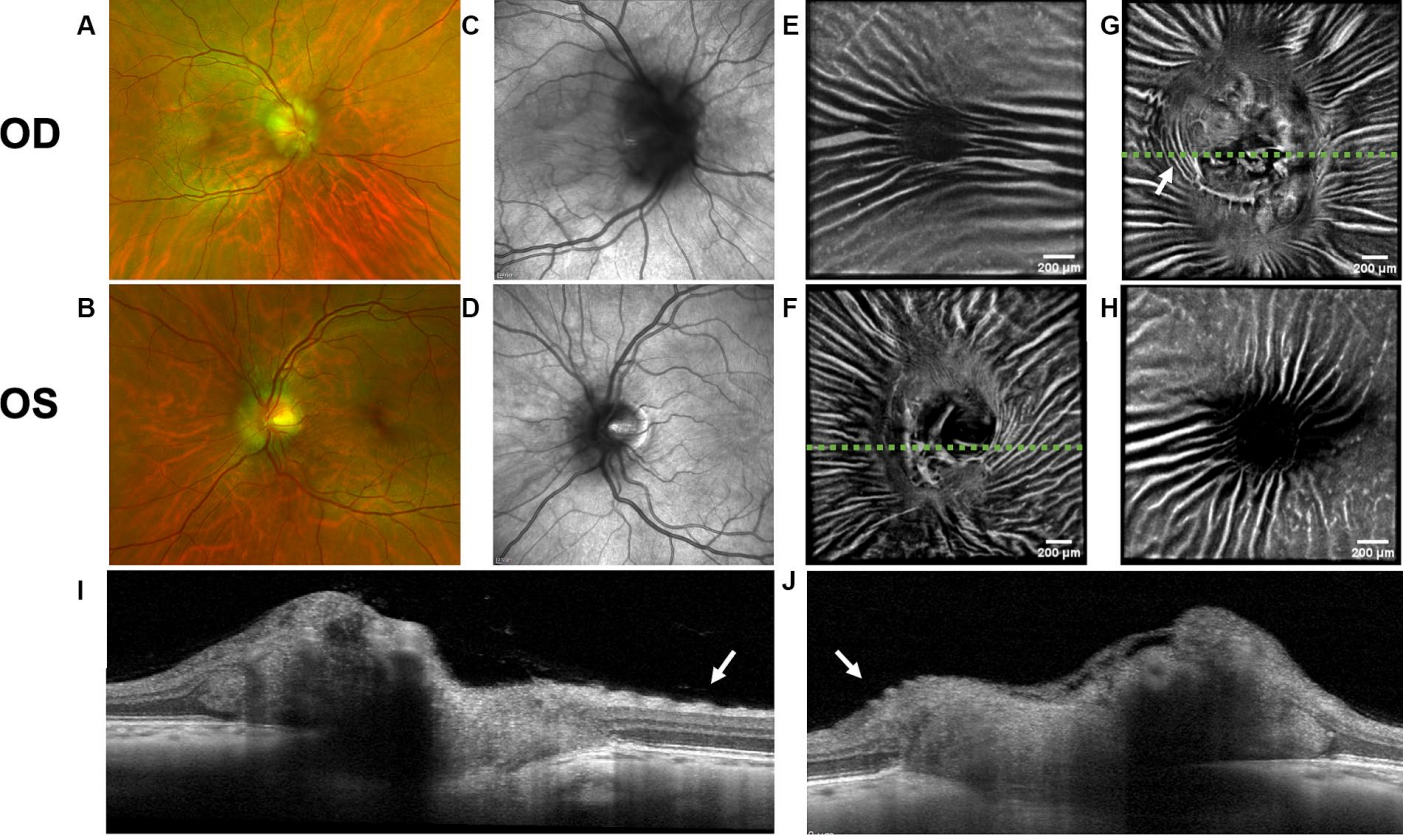
Retinal imaging of subject 4 with IIH. **(A)** Color fundus photo OD. **(B)** Color fundus photo OS. **(C)** SLO image of the OD ONH. **(D)** SLO image of the OS ONH. **(E)** Averaged en-face OCT scan of the OD macula. **(F)** Averaged en-face OCT scan of the OS ONH. The green dashed line indicates the transverse position of the B-scan in image **(I)**. **(G)** Averaged en-face OCT scan of the OD ONH with concentric PPW (white arrow) and radial RF. The green dashed line indicates the transverse position of the B-scan in image **(J)**. **(H)** Averaged en-face OCT scan of the OS macula. **(I)** EDI-OCT B-scan of the OS ONH with retinal folds (white arrow). The green dashed line in image **(F)** indicates the transverse position on the en-face image. **(J)** EDI-OCT B-scan of the OD ONH with PPW (white arrow). The green dashed line in image **(G)** indicates the transverse position on the en-face image, where the same PPW are indicated with a white arrow.

**Figure 4:**
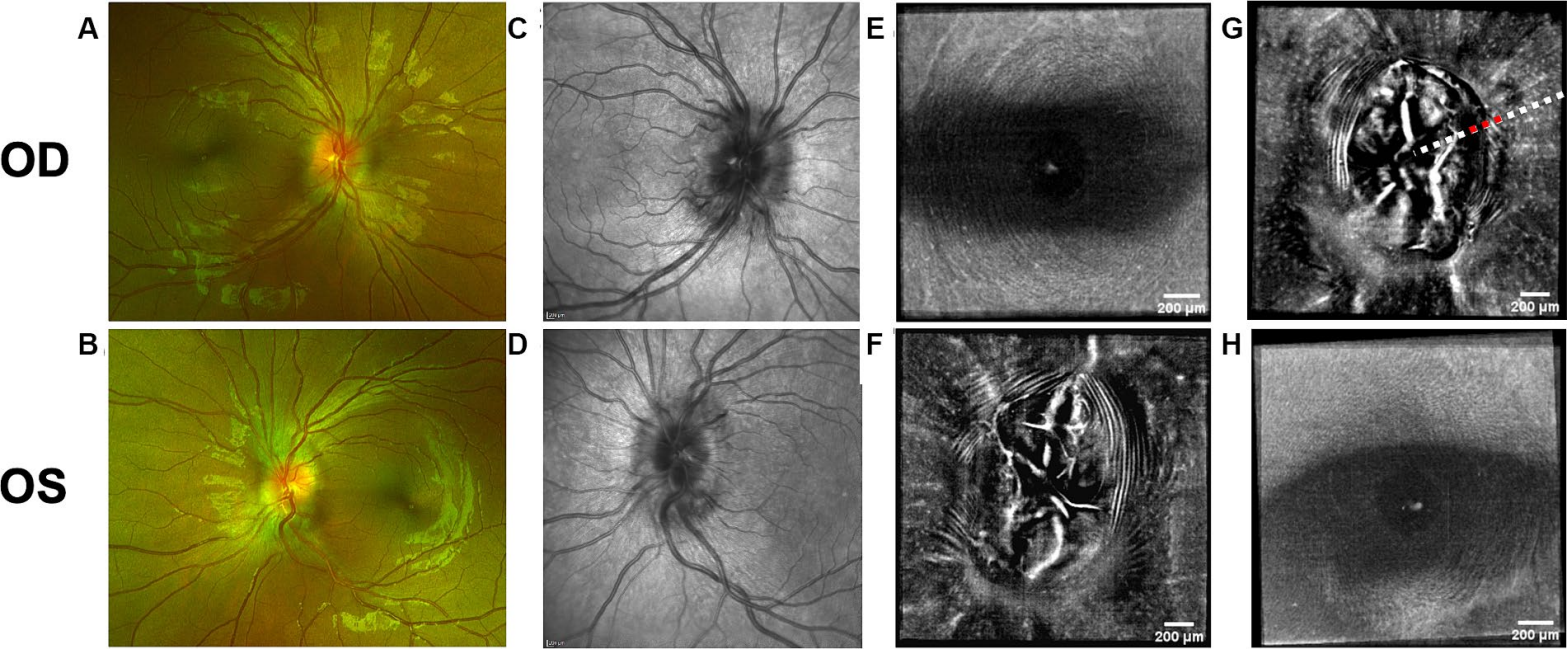
Retinal imaging of subject 5 with secondary pseudotumor cerebri syndrome. **(A)** Color fundus photo OD. **(B)** Color fundus photo OS. **(C)** SLO image of the OD ONH. **(D)** SLO image of the OS ONH. **(E)** Averaged en-face OCT scan of the OD macula. **(F)** Averaged en-face OCT scan of the OS ONH. **(G)** Averaged en-face OCT scan of the OD ONH. The red dashed line indicates the location of B-scan negative PPW on the transverse B-scan, whose location is indicated by the white dashed line. **(H)** Averaged en-face OCT scan of the OS macula.

**Figure 5:**
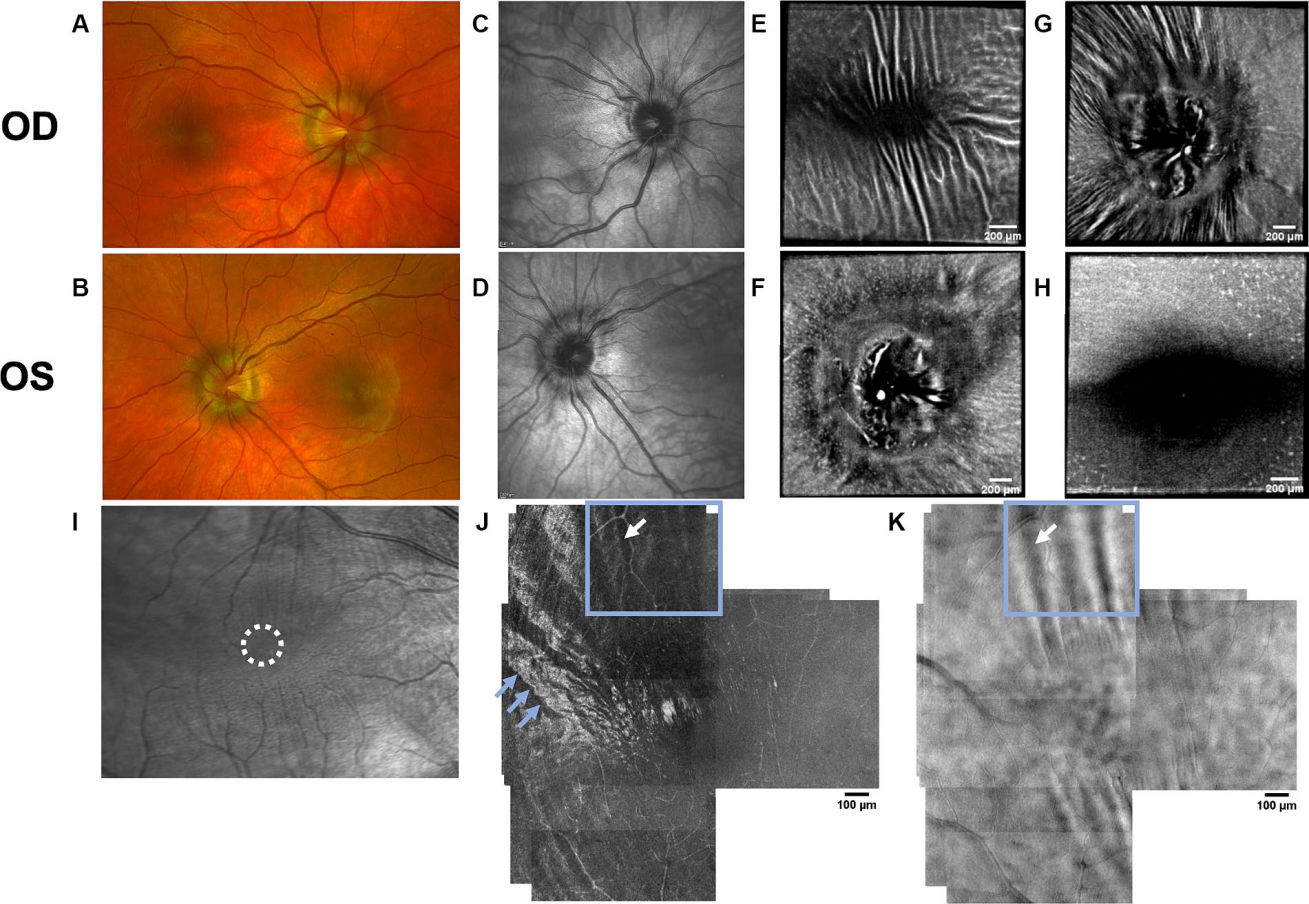
Retinal imaging of subject 6 with IIH. **(A)** Color fundus photo OD. **(B)** Color fundus photo OS. **(C)** SLO image of the OD ONH. **(D)** SLO image of the OS ONH. **(E)** Averaged en-face OCT scan of the OD macula. **(F)** Averaged en-face OCT scan of the OS ONH. **(G)** Averaged en-face OCT scan of the OD ONH. **(H)** Averaged en-face OCT scan of the OS macula. **(I)** SLO image of the OD macula with RF. The region where the AOSLO images **(J,K)** were captured is marked with a white circle. **(J)** Confocal AOSLO image of the OD temporal fovea with a 2-degree field of view. RF are seen as hypo- and hyperreflective bands (white arrow in blue box). The hyperreflective banding temporally (blue arrows) are an interesting feature without known cause, as it cannot be seen in other modalities. The region where the AOSLO image was captured is marked with a white circle in image **(I)**. **(K)** AOSLO non-confocal split detection image of the OD temporal fovea with a 2-degree field of view. RF are seen (white arrow and blue box). The region where the AOSLO image was captured is marked with a white circle in image **(I)**.

**Figure 6:**
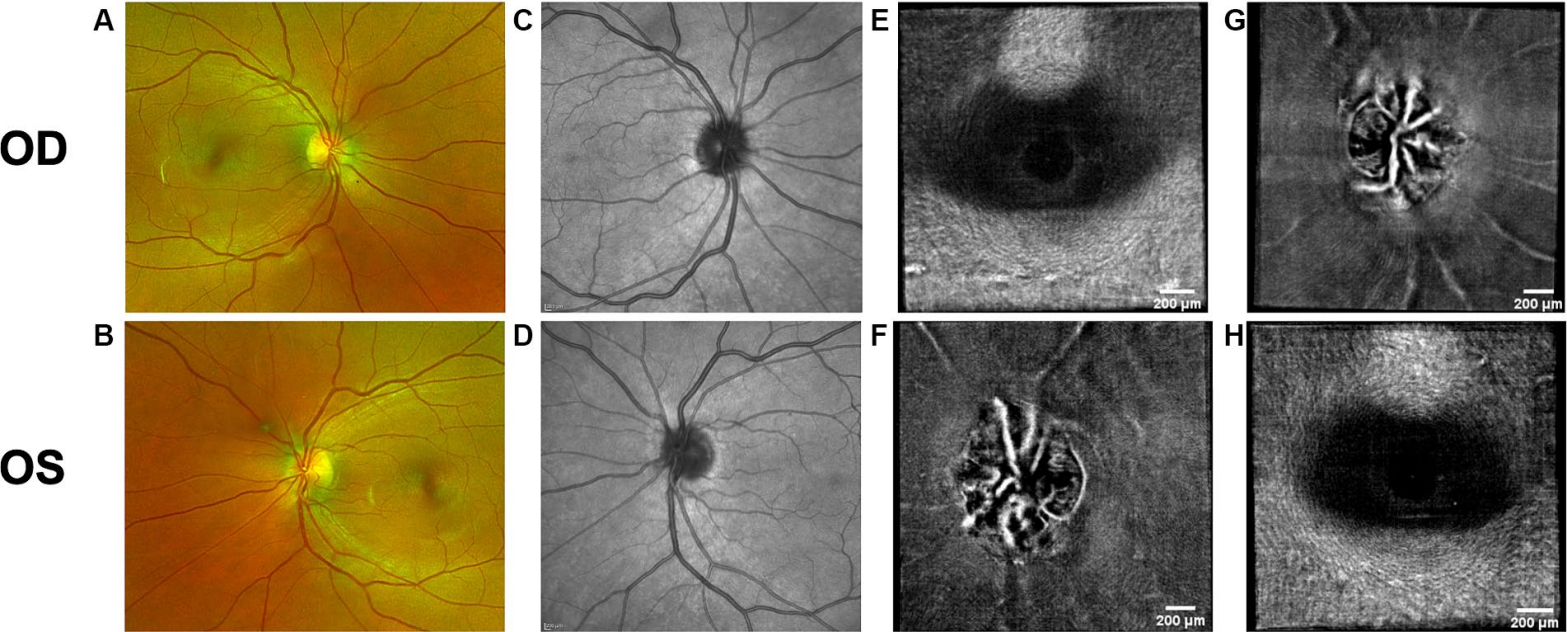
Retinal imaging of subject 2 with IIH. **(A)** Color fundus photo OD. **(B)** Color fundus photo OS. **(C)** SLO image of the OD ONH. **(D)** SLO image of the OS ONH. **(E)** Averaged en-face OCT scan of the OD macula. **(F)** Averaged en-face OCT scan of the OS ONH. **(G)** Averaged en-face OCT scan of the OD ONH. **(H)** Averaged en-face OCT scan of the OS macula.

**Figure 7:**
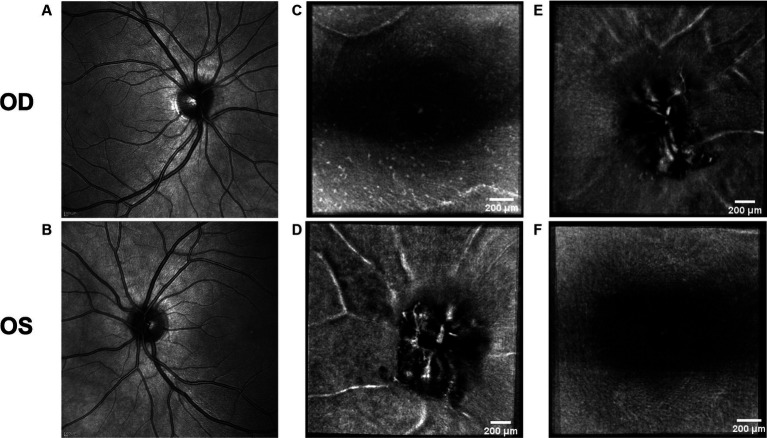
Retinal imaging of the control subject. **(A)** SLO image of the OD ONH. **(B)** SLO image of the OS ONH. **(C)** Averaged en-face OCT scan of the OD macula with visible hyalocytes. **(D)** Averaged en-face OCT scan of the OS ONH. **(E)** Averaged en-face OCT scan of the OD ONH. **(F)** Averaged en-face OCT scan of the OS macula.

**Figure 8:**
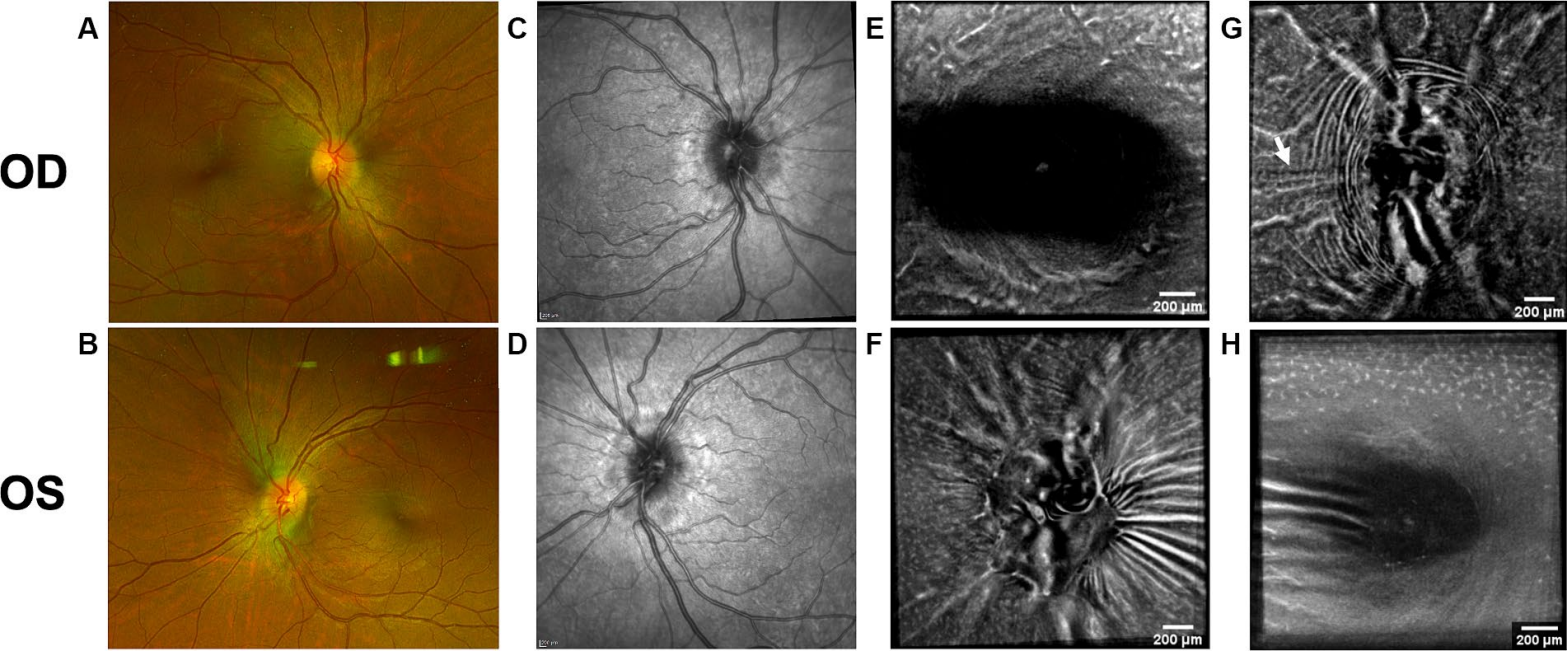
Follow-up retinal imaging of subject 3 with IIH 2 ½ months after baseline imaging. **(A)** Color fundus photo OD. **(B)** Color fundus photo OS. **(C)** SLO image of the OD ONH. **(D)** SLO image of the OS ONH. **(E)** Averaged en-face OCT scan of the OD macula. **(F)** Averaged en-face OCT scan of the OS ONH. **(G)** Averaged en-face OCT scan of the OD ONH with regressed horizontal retinal folds in the papillomacular bundle (white arrow) compared to Figure 2G. **(H)** Averaged en-face OCT scan of the OS macula.

Both PPW and RF were visible with one or more imaging modalities in 9 of 10 eyes with papilledema at baseline (Table 4; Figures 1–5), but neither the high ICP eyes without papilledema nor the control eyes (Table 4; Figures 6, 7). Both RF and PPW were also present bilaterally in subject 3 at follow-up imaging (Figure 8). RF and PPW were more pronounced in the temporal region compared to the nasal region on both B-scans and averaged en-face OCT scans. We were able to observe a slight reduction in RF in the follow-up images of subject 3 [Figures 2G and 8G (white arrow)] at imaging after 2 ½ months from baseline.

**Table 4:** Peripapillary wrinkles and retinal folds.

Subject	1		2		3 (Baseline)		3 (Follow-up)		4		5		6		Control	
	(Figure 1)		(Figure 6)		(Figure 2)		(Figure 8)		(Figure 3)		(Figure 4)		(Figure 5)		(Figure 7)	
	OD	OS	OD	OS	OD	OS	OD	OS	OD	OS	OD	OS	OD	OS	OD	OS
**OCT B-scans**																
PPW	X	X	–	–	X	X	X	X	X	X	X	X^1^	X	–	–	–
Disc RF	X	X^(*)^	–	–	X	X	–	X	X	X	X^*^	X^1*^	X	–	–	–
Macula RF	–	–	–	–	–	–	–	–	X	X	–	–	X	–	–	–
**Averaged en-face OCT scans**																
Concentric PPW	X	X	–	–	X	X	X	–	X	–	X	X	–	–	–	–
Spiral PPW	X	X	–	–	X	X	–	X	–	X	X	X	X	–	–	–
Disc RF	X	X	–	–	X	X	X	X	X	X	–	–	X	–	–	–
Macula RF	–	–	–	–	X	X	–	X	X	X	–	–	X	–	–	–
**OCT B-scan negative**																
PPW	X	–	–	–	X	X	X	X	–	–	X	Cannot be determined	X	–	–	–
Disc RF	–	X	–	–	X	X	X	X	X	X	–	Cannot be determined	X	–	–	–
Macula RF	–	–	–	–	X	X	–	X	–	–	–	–	–	–	–	–
**Fundus photo**																
PPW	–	–	–	–	–	–	–	–	–	X	–	–	–	–	–	–
Disc RF	–	–	–	–	–	–	–	–	X	X	–	–	–	–	–	–
Macula RF	–	–	–	–	–	–	–	–	–	X	–	–	–	–	–	–
**AOSLO**																
PPW	N/A	–											N/A	N/A		
RF	–	N/A											X	N/A		

The table shows the presence of PPW and RF on the AOSLO images, OCT B-scans, the averaged en-face OCT scans as well as the presence of OCT B-scan negative PPW and RF (PPW/RF seen on averaged en-face OCT scans but not on the B-scans). The same averaged en-face OCT image can have both B-scan positive PPW/RF, as well as B-scan negative PPW/RF. “X” marks the presence of the structures. ^*^Only peripapillary intraretinal folds. (*) Including peripapillary intraretinal folds. ^1^Only evaluated on raster scan. OD, Right eye; OS, Left eye; OCT, Optical coherence tomography; PPW, Peripapillary wrinkles; RF, Retinal folds; AOSLO, Adaptive optics scanning light ophthalmoscopy.

OCT B-scans were less sensitive to PPW and RF compared to the averaged en-face OCT scan, with 8 eyes (at baseline) having OCT B-scan negative RF and/or PPW. Reasons for OCT B-scan negative RF and/or PPW included scan orientation, scan area and scan resolution. For example, subject 4 had many RF on the averaged en-face OCT scan of the optic disc, which were not visible on the EDI-OCT B-scan (Figure 3; Table 4). On the averaged en-face OCT image, it was also easier to classify the type of PPW and RF present compared to the B-scans, apart from the peripapillary intraretinal folds, which were only visible on the latter. Concentric and spiral PPW as well as radial, oblique and horizontal RF could be easily identified on the averaged en-face OCT-scan. Fundus photos were not sensitive to detection of PPW and RF, only detecting them in one subject (subject 4; Figure 3; Table 4).

As on the averaged en-face OCT images and B-scans, we were also able to visualize radial RF on the AOSLO non-confocal split detection image of subject 6’s right temporal fovea. They were also present on the confocal image, but not as evident (Figures 5J,K). Temporally on the confocal image we were able to determine hyperreflective banding (Figure 5J), which did not seem to correspond to folds on the non-confocal split detection image (Figure 5K). Furthermore, they did not appear to have corresponding features on the SLO scan (Figure 5I) or OCT B-scan of the macula. In subject 1, who also had high ICP, PPW/RF were only visible on the averaged en-face OCT images and OCT B-scans, but not on the AOSLO images (Figures 1F,G,K,L,M).

### 3.4 Peripapillary hyperreflective ovoid mass-like structures

A PHOMS was present in both eyes of all subjects with papilledema at baseline (5 subjects, 10 eyes) as either a full or a partial torus around the optic disc (Table 1), but not in the subject without papilledema or in the control subject. PHOMS were also present bilaterally in subject 3 at follow-up imaging. In severe cases of papilledema, the margins of PHOMS could not consistently be visualized and therefore could not be measured in all scans [subject 3 (baseline), 4 and 5]. In other cases, PHOMS were shadowed by vessels and therefore could not be measured. In the 4 subjects with measurable PHOMS at baseline, the median PHOMS volume of the 8 eyes was 1.37 mm^3^ (range 0.91–2.78). The median of the BMO diameter in the same 8 eyes was 1607.08 (range 1418.00–1691.33). The median of the BMO diameter in the 4 eyes without PHOMS (subject 2 and the control) was 1350.17 (range 1285.83–1483.00).

The sample size precludes meaningful quantification of the PHOMS and Frisén grade relationship. However, in certain subjects Frisén grade and PHOMS volume were not closely correlated. For example, subject 1 OD and subject 4 OD had similar PHOMS volume, yet subject 4 OD had a higher Frisén grade (Table 1).

AOSLO imaging was performed close to the optic disc on subject 1 in an attempt to visualize axoplasmic stasis/PHOMS. There has in a previous study been reported micron-size hyperreflective spots in RGC axons, which are thought to indicate axoplasmic movement (27). This was not visualized in our subject. However, it is not known if this could be due to reduced axoplasmic movement due to axoplasmic stasis, image quality or a different cause. Imaging of the temporal peripapillary area of the subject’s right eye (Figure 1K) was unremarkable. Imaging of the temporal/inferotemporal peripapillary area of the left eye (closer to the disc than the right eye) showed features that had a cellular appearance on both confocal and non-confocal split detection images (Figures 1L,M). The hyperreflective dots visualized in the confocal image (Figure 1L) do not share the same characteristics as the hyperreflective structures reported in other publications as Gunn’s dots (37). We hypothesize that they could either be part of a normal retina or a pathological feature, associated with papilledema, not previously identified. Further study is required to determine their basis and relevance to papilledema and high ICP.

### 3.5 Hyalocytes

Hyalocytes were visible in all high ICP subjects except subject 2, either at the macula, optic disc or at both locations (Figures 1–8). They were also observed in the control subject. More hyalocytes were generally observed around the disc than at the macula. However, it was not possible to quantify as well as evaluate the uniformity, symmetry, density and distribution of hyalocytes in most images due to PPW, RF and vessels preventing accurate assessment. For example, in subject 4 it was not possible to identify any hyalocytes around the ONH as PPW and RF covered the entire imaged area (Figures 3F,G). This was also the case in subject 1, 3, 5 and 6, where the beforementioned structures affected their visualization (Figures 1, 2, 4, 5, 8). The density of hyalocytes does not appear to relate with the severity of papilledema or the presence of high ICP as subject 6 had less severe papilledema than subject 3 but a denser distribution of hyalocytes (Figures 2, 5). In the control, hyalocytes were present despite the subject not having papilledema or elevated ICP (Figure 7).

### 3.6 Image acquisition and processing time

6 of 7 subjects underwent pupillary dilation for the OCT scans (Spectralis and OptoVue). All subjects undergoing AOSLO imaging were dilated.

Of the three modalities (Table 5) structural OCT (Spectralis) had the shortest imaging and image processing time. The scans in the protocol provided the conventional measurements, which were supplemented by the custom PHOMS measurements. The full imaging protocol for the averaged en-face OCT images required 2–3 times longer to complete than structural OCT due to the multiple volume scans collected. The processing of these images was also more time consuming than processing OCT B-scans. For both OCT protocols one of the major challenges was severe papilledema, which made it difficult to obtain a good quality full view of the optic nerve head on the scan.

**Table 5:** Comparison of imaging modality acquisition and processing time.

	OCT	Averaged en-face OCT	AOSLO
Imaged subjects	All subjects	All subjects	1, 6 and control
Dilation time	20 min	20 min	20 min
Personnel needed for acquisition	1	1	2
Imaging time (total)	30–40 min	60–90 min	30–60 min
Processing time (1 person)	30 min/eye	60 min/eye/location^*^	Up to 80 h per eye
Processing technique	Manual and automatic	Manual and automatic	Manual
Challenges	Severe papilledema, dry cornea, fixation, fatigue	Severe papilledema, dry cornea, fixation, fatigue	Accommodation, dry cornea, fixation, fatigue, light reflection, anatomy of the eye, scheduling of experienced personnel

^*^Location: Macula and disc. OCT, Optical coherence tomography; AOSLO, Adaptive optics scanning light ophthalmoscopy.

Lastly, the custom-built AOSLO was the most time-consuming imaging modality even for an experienced team – both in regards to imaging and image processing (Table 5). Challenges especially related to AOSLO imaging are light reflection and fixation. This was seen in subject 1, as good images close to the optic disc were more challenging to obtain compared to further from the disc due to poor light reflection in this area. In subject 6 good images of the macula were also difficult to obtain due to the patient’s poor fixation and reduced tear film. In both patients the anatomy of the eyes could also have played a role in the difficulty of obtaining optimal images.

## 4 Discussion

There is an unmet need for non-invasive surrogate biomarkers for ICP. Many ophthalmic structural changes are candidate biomarkers because they can be assessed non-invasively with high resolution. Our aim was to assess retinal structural changes through novel multimodal imaging in patients with elevated ICP to further characterize some of these biomarkers and assess their candidacy. The implementation of several modalities in this exploratory study on ICP surrogate measures allowed us to gain further insight into both novel and known ophthalmic structural changes and their potential to convey information regarding changes to the visual afferent pathway. Furthermore, we assessed their feasibility for implementation in research and clinical settings.

Previous studies have established RF and PPW as common findings in patients with optic disc edema (38, 39). They are classically determined using OCT and fundus images. Studies have shown that OCT has a higher sensitivity of visualizing RF, PPW and choroidal folds (CF) compared to fundus imaging – specifically using OCT B-scan raster and en-face imaging (15) and we made a similar observation. However, by averaging en-face OCT scans, which increases the signal-to-noise ratio, we were able to improve visualization of the folds and wrinkles in our patients beyond SLO and OCT B-scan images (Figures 1–5, 8). On the OCT B-scans the RF and PPW must be oriented perpendicular to the transverse axial scan in order to be visible, which means that not all PPW and RF are visible in the B-scans and therefore the severity of the condition is not as evident as it is on the averaged en-face OCT scans. This was seen in 5 subjects (Table 4), for example on the scans of the ONH bilaterally on subject 4 (Figure 3). A limitation of the macular averaged en-face OCT scans is that we see a smaller area of the macula than on the conventional OCT B-scans and commercial SLO scan of the macula. We believe this imaging approach could supplement current techniques for evaluating PPW and superficial RF. While intraretinal folds are not visualized in our protocol, it may be possible to use en-face segmentation of deeper layers to achieve this.

A previous study observed that RF diminish with treatment of ICP, coincident with reduction of RNFL thickness and ONH swelling at 6 months, where PPW resolved over a longer period (15, 39). We observed this in our single subject with follow up imaging (Figures 2G, 8G). Averaged en-face OCT may make it easier to follow the progression of these structures within an individual, as we detected OCT B-scan negative PPW/RF in several subjects (Table 4). Furthermore, the averaged en-face OCT images can also be helpful in visualizing the types and patterns of PPW and RF, which may vary due to the etiology of optic disc edema as well as anatomical and material property differences between people, as studies have shown a difference in PPW and RF patterns in non-arteritic ischemic optic neuropathy (NAION) compared to IIH (39). Furthermore, the imaging technique could provide insights into the biomechanical differences of the pathology in optic disc edema as PPW and RF are seen to be associated with higher grades of papilledema (i.e., greater mean Frisén grade, ONH volume, RNFL thickness and optic disc height), whereas CF (as well as RF) are associated with anterior deformation of the peripapillary retinal pigment epithelium/basal membrane layer. CF have also been associated with higher levels of ICP (15, 39).

RF were also visualized on the AOSLO montage of the fovea of subject 6 (Figure 5). Though this finding did not give a better overview of the condition than averaged en-face OCT scans, it is scientifically interesting due to the potential to simultaneously investigate how the RF and PPW impact the NFL on a cellular level. Neither RF nor PPW have been characterized before using this imaging platform.

Folds near the disc have not been associated with vision loss (MD or VA) in patients with mild visual impairment (15, 40). However, patients with IIH and mild visual field loss have been found to have reduced high-contrast VA in the eyes with persistent macula folds (40). We could confirm in the present study that PPW and RF did not significantly affect visual function based on VA and HVF MD in this cohort of subjects. However, our sample did not cover a broad enough range of visual impairments to permit correlation analysis. Therefore, it would be interesting to investigate this correlation among patients with moderate to severe vision loss.

A PHOMS is an OCT-specific marker of axoplasmic stasis (17). We investigated the presence and volume of PHOMS using OCT to elucidate the consequences of axoplasmic stasis on other structural and functional changes of the retina and optic nerve in papilledema. The subjects in this study had normal VA and normal to mild vision loss, based on MD, despite having a large PHOMS volume, indicating that PHOMS does not prominently affect visual function in papilledema, at least not in the initial stages of the papilledema.

In this study, subject 4 had moderate/severe papilledema in the right eye with a relatively high RNFL thickness and large PHOMS volume (Tables 1, 3). The right eye of subject 1 had a relatively high RNFL thickness and a large PHOMS volume yet a low Frisén grade. Regression of PHOMS has been shown to be associated with regression of papilledema (17, 41, 42), yet previous research shows lack of correlation between PHOMS and RNFL thickness (43). Despite, these three measures seeming complementary, further research is needed to understand how they might be used together to inform clinical care of patients with papilledema. It should be noted that Frisén grade is subjective (12) as opposed to PHOMS volume and RNFL thickness. Further research comparing these values over time is needed to validate a correlation between them.

BMO is slightly larger in the eyes with PHOMS compared to the eyes without, however, this might be an artifact of shadowing, which can be a barrier to accurate identification of the Bruch’s membrane margin.

A strength of PHOMS volume as a biomarker is that many clinics already have the technology required to obtain the necessary images, yet, there are limitations in the lack of automated measurements and imaging artifacts (e.g., shadowing due to moderate/severe papilledema) precluding accurate measurements in all subjects and scans. Another limitation to PHOMS as a biomarker is lack of specificity since PHOMS can also be seen in several other conditions for example with optic disc drusen and crowded or tilted discs (42).

Hyalocytes were visualized using averaged en-face OCT images. These might be a marker of retinal injury based on previous studies (21), though our visualization of hyalocytes in subjects with IIH/SPTC as well as in the control subject does not support this. Further studies of hyalocyte density, distribution and symmetry in papilledema subjects were limited as other retinal changes in IIH/SPTC, namely PPW, RF and vessels, prevented their consistent visualization [e.g., Subject 3 (baseline); Figures 2E–H].

The aberrations of the optics of the eye limit the resolution of conventional ophthalmic imaging such as OCT. In the present study we used a custom-built AOSLO ophthalmoscope, which diminishes aberrations of the eye, to conduct high-resolution imaging of the nerve fiber bundles close to the optic disc and macula to identify potentially pathological structural changes in the subjects with IIH/SPTC and compare these to other imaging techniques. As mentioned, previous studies have discovered micron-size hyperreflective spots along the nerve bundles on patients with glaucoma, which were thought to be indicative of axonal transport (27), however AO imaging has not previously been applied to eyes with optic disc edema. In the right eye of subject 1 we captured images in the peripapillary area, which had unremarkable findings including no hyperreflective spots suggestive of axonal transport. In the left eye there were novel features of unclear etiology, which would be interesting to study further in a larger patient cohort with elevated ICP to evaluate their correlation to ICP. This technology could be the next step to determine a biomarker of elevated ICP in patients, who do not show macroscopic retinal changes (e.g., subject 2). We cannot draw conclusions about the relationship between AOSLO features and vision loss since our imaged subjects had preserved or mildly impaired vision and our sample was small.

Limitations to this study include the small sample size, as well as only having 1 control. Therefore, quantitative comparisons are precluded. Quantitative longitudinal measurements in a larger cohort might show other patterns and changes over time. Additionally, all of our subjects had normal vision or minimal vision loss, so we cannot extrapolate to patients with more severe vision loss. This study captured a cross-section of subjects who were imaged at different times in their treatment process and who had different responses to elevated ICP. This variability is furthermore emphasized by the anatomical variation between patients and between eyes in the individual patient, which plays a role in the severity of papilledema (44). None the less the comparison between imaging modalities within eyes provides important insights in how they might complement each other to guide management of patients with IIH/SPTC.

The purpose of this research was to explore candidate ocular biomarkers of elevated ICP using multimodal non-invasive ophthalmic imaging platforms. Establishing a non-invasive easily accessible biomarker can reduce the time, cost and discomfort for patients. This pilot study investigated three main structural changes of the retina thought to be potentially caused by elevated ICP—PPW/RF, PHOMS, and hyalocytes. All three structures were present in subjects with papilledema, suggesting an association with elevated ICP. However, we did not elucidate sensitivity or specificity. Further research is needed to validate if the studied structures can be reliable biomarkers. The density, distribution, and symmetry of hyalocytes is unlikely to be a useful biomarker due to image distortion by PPW and RF precluding accurate assessment in subjects with papilledema. OCT imaging of PPW, RF and PHOMS volume as well as AOSLO for studying PPW, RF and new features due to elevated ICP, could have scientific interest for investigating the pathophysiology of papilledema and the possible occurrence of vision loss in patients with moderate to severe vision impairment at diagnosis. A future perspective for further research is also to compare structural retinal changes (e.g., PHOMS, papilledema, RF and PPW) in subjects *with* and *without* elevated ICP. This would give us insight into how ICP affects the structural patterns and pathology of the retina in these patients, which would be a very beneficial clinical tool in order to potentially eliminate measuring LOP unnecessarily. However, there is a need to investigate if the image acquisition and processing protocols can be shortened or automated, as the protocols currently are too time consuming to be implemented in a clinical setting. Artificial intelligence using deep learning algorithms are emerging fields within ophthalmic imaging (45, 46). Creating a central repository for OCT scans would allow researchers to develop this technology and aid doctors in determining the presence of retinal structures more accurately and their relevance in clinical care of patients.

## Data availability statement

The raw data supporting the conclusions of this article will be made available by the authors, without undue reservation.

## Ethics statement

The studies involving humans were approved by the Institutional Review Board at Stanford University. The studies were conducted in accordance with the local legislation and institutional requirements. The participants provided their written informed consent to participate in this study.

## Author contributions

MG-N: Conceptualization, Investigation, Project administration, Writing – original draft, Writing – review & editing, Formal analysis. AD: Writing – review & editing, Conceptualization, Data curation, Investigation, Resources. RD: Conceptualization, Writing – review & editing, Investigation. SH: Conceptualization, Writing – review & editing, Methodology, Writing – original draft. HM: Conceptualization, Funding acquisition, Investigation, Methodology, Project administration, Resources, Supervision, Writing – original draft, Writing – review & editing.
