# Supplementary material for: Application of novel non-invasive ophthalmic imaging to visualize peripapillary wrinkles, retinal folds and peripapillary hyperreflective ovoid mass-like structures associated with elevated intracranial pressure
Source: Front Neurol. 2024 Jun 18;15:1383210. doi: 10.3389/fneur.2024.1383210 (PMC11217179; doi:10.3389/fneur.2024.1383210)

Supplementary figures

Supplementary figure 1 | Measurements of the optic nerve head

The central optic nerve head (ONH) volume (central red number = 0.70) and central ONH thickness (central black number = 888) were extracted within the 1 mm diameter circle. Maximum height centrally in the ONH (right column, *"central max"* = 1194).

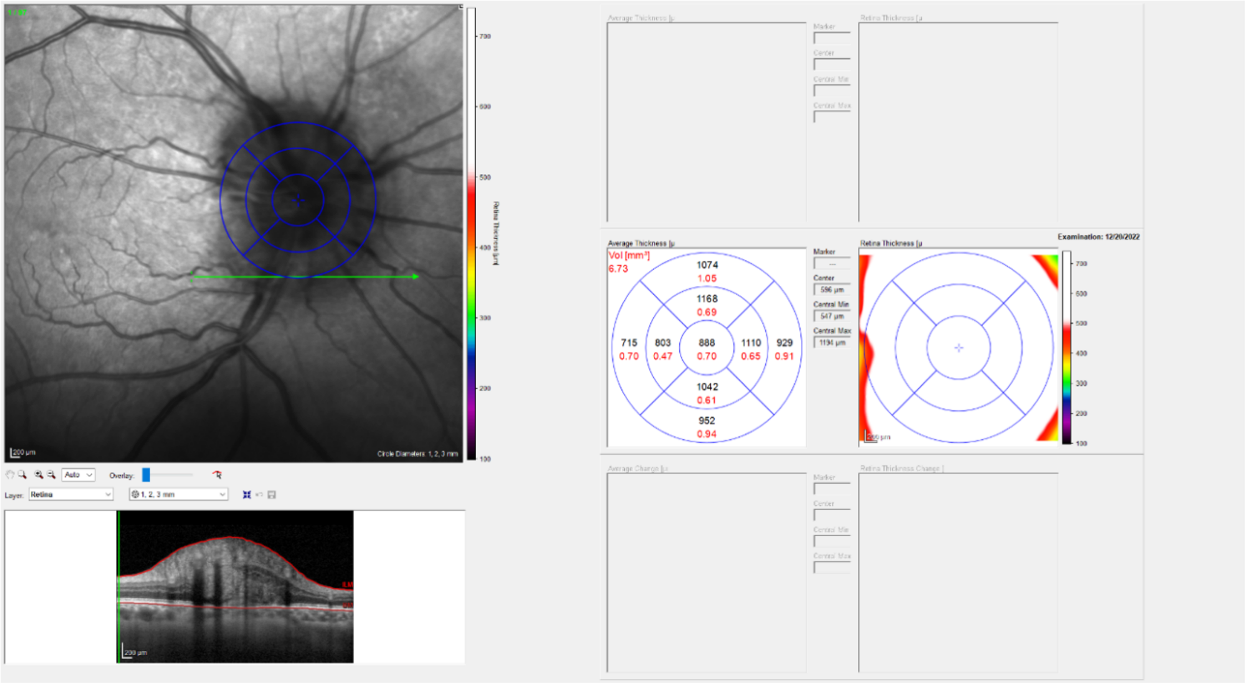

Supplementary figure 2 | PHOMS measurements

Measurements on an EDI-OCT B-scan of the peripapillary hyperreflective ovoid mass-like structure (PHOMS) on the right optic nerve head of subject 1. b = horizontal cross-sectional radius of the PHOMS. c = the vertical cross-sectional radius of the PHOMS. BMO = the diameter of the Bruch's membrane opening. w = the distance between the edge of the BMO and the intersection between b and c.

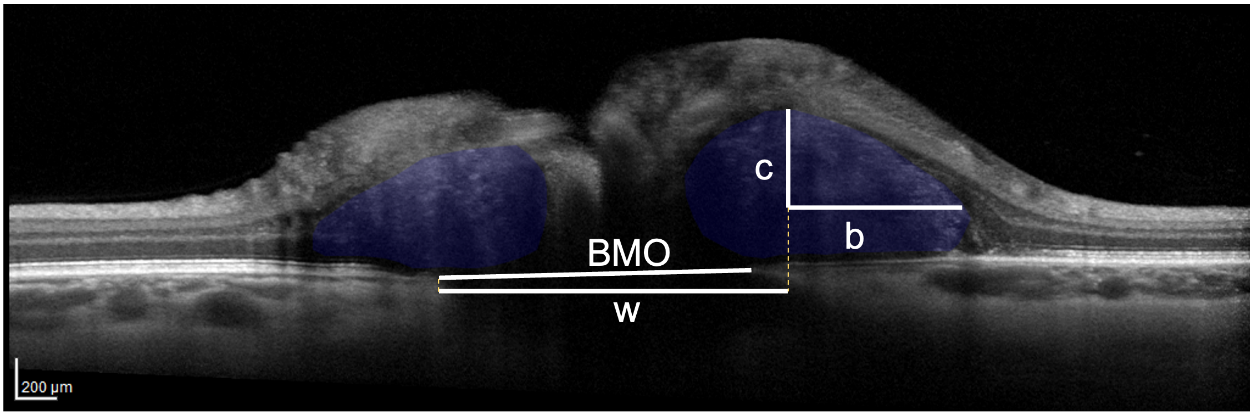

Supplementary figure 3 | Optic disc drusen
EDI-OCT B-scan of the OD ONH of subject 2. The yellow arrow indicates an optic disc drusen. This could affect the peripapillary RNFL in this subject, as drusen are seen to thicken the RNFL.

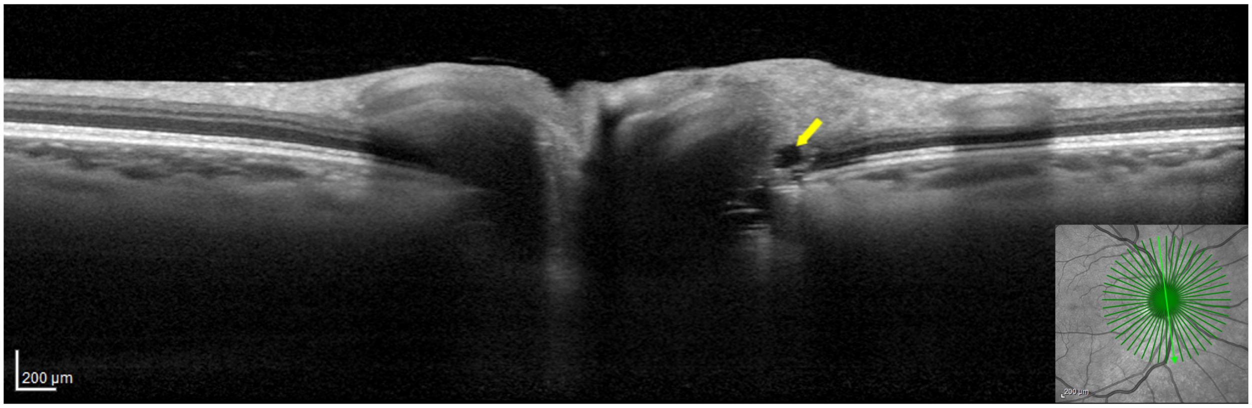

Supplement: Supplementary file 1 [file Data_Sheet_1.pdf]
